# Treatment challenges in co-occurring substance use and gambling disorder: A case report

**DOI:** 10.1192/j.eurpsy.2025.971

**Published:** 2025-08-26

**Authors:** E. Medved, M. Delić

**Affiliations:** 1 University psychiatric clinic Ljubljana, Ljubljana, Slovenia

## Abstract

**Introduction:**

Gambling disorder was previously classified as an impulse control disorder in the DSM-IV, but with the introduction of DSM-5, it has been redefined as a behavioural addiction. This reclassification reflects a growing understanding of how gambling shares many characteristics with substance use disorders, including the compulsive nature of the behaviour, its neurological underpinnings, and the significant consequences it has on an individual’s life. The shift from an impulse disorder to an addiction framework highlights the chronic, relapsing nature of the condition and underscores the need for more comprehensive treatment approaches. Gambling disorder is now recognized as one of the major public health issue due to its association with significant psychological, social, and financial harm. Furthermore, it frequently co-occurs with other psychiatric conditions, particularly mood disorders and substance use disorders, making treatment and recovery more complex. This dual diagnosis often results in overlapping symptoms that reinforce each other, complicating the course of treatment and decreasing the likelihood of sustained abstinence. Addressing the multifaceted nature of gambling disorder is critical to improving therapeutic outcomes, particularly in individuals with comorbid conditions.

**Objectives:**

This case report aims to examine the challenges in maintaining abstinence in individuals with co-occurring substance use and gambling disorders, focusing on the role of impulsivity and motivational instability. It also seeks to explore effective treatment strategies to improve long-term recovery outcomes.

**Methods:**

A case report approach was employed, following a 23-year-old male patient with a history of multiple psychiatric hospitalizations due to substance use and gambling disorders. The patient’s treatment journey was analysed, with particular attention to his motivation, therapeutic engagement, and relapse patterns.

**Results:**

The patient struggled with maintaining long-term abstinence due to impulsivity, frustration intolerance, and repeated relapses, exacerbated by non-compliance with treatment. Although initial motivation for recovery was present, it deteriorated over time, resulting in premature termination of treatment programs. Persistent gambling and substance use led to significant personal and financial consequences.

**Conclusions:**

Effective treatment for co-occurring gambling and substance use disorders must prioritize enhancing frustration tolerance, impulse control, and stable motivation. Comprehensive therapeutic interventions, continuous support, and realistic goal-setting are crucial for improving abstinence and preventing relapse. Understanding the interconnectedness of gambling and substance use is key to tailoring effective treatments.

**Disclosure of Interest:**

None Declared

